# Supplementary material for: Hematopoietic stem cells with granulo-monocytic differentiation state overcome venetoclax sensitivity in patients with myelodysplastic syndromes
Source: Nat Commun. 2024 Mar 18;15:2428. doi: 10.1038/s41467-024-46424-3 (PMC10948794; doi:10.1038/s41467-024-46424-3)
Supplement: Supplementary file 1 — Supplementary Information [file 41467_2024_46424_MOESM1_ESM.pdf]

Supplementary Figure 1

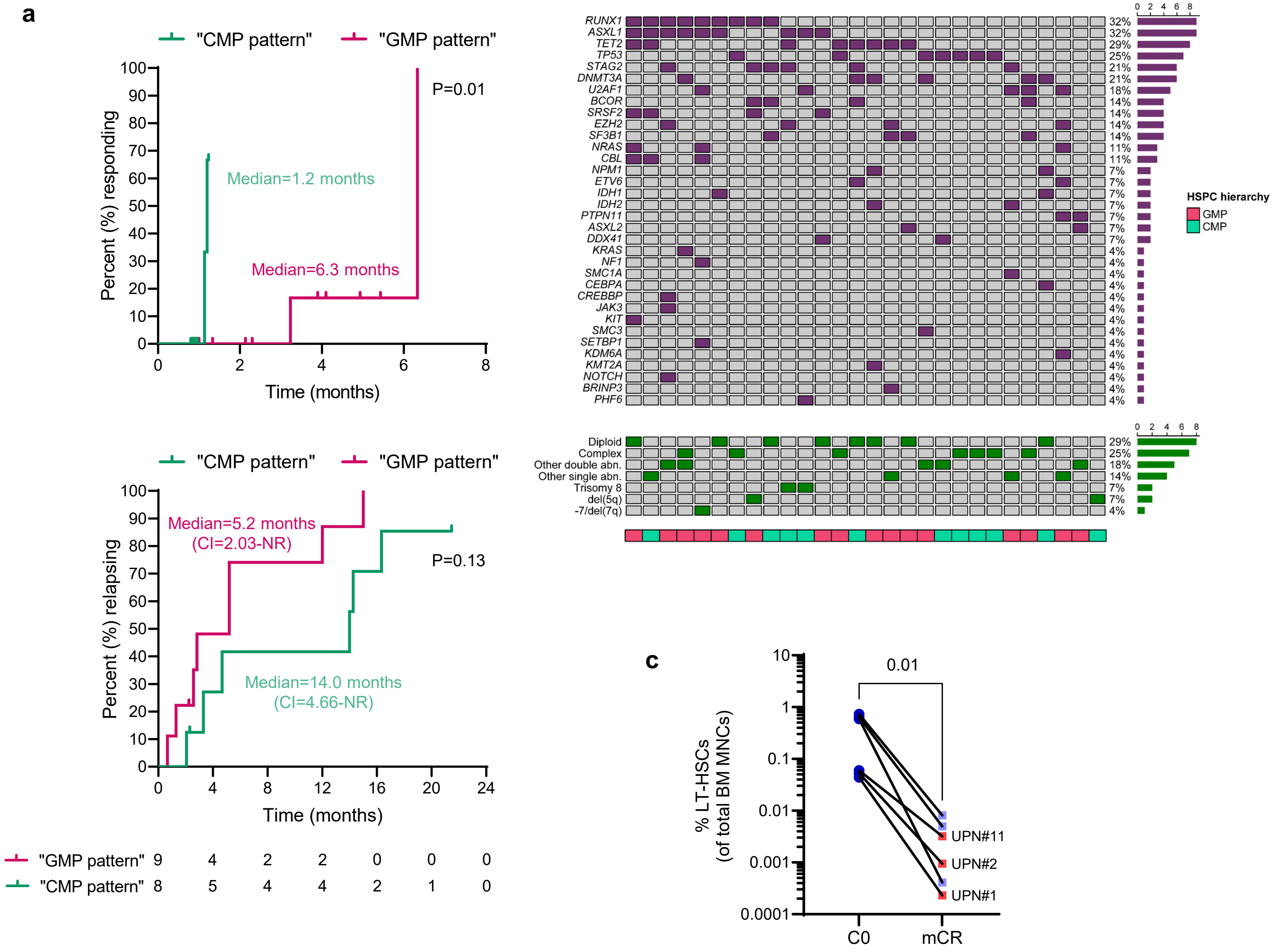

Supplementary Figure 2

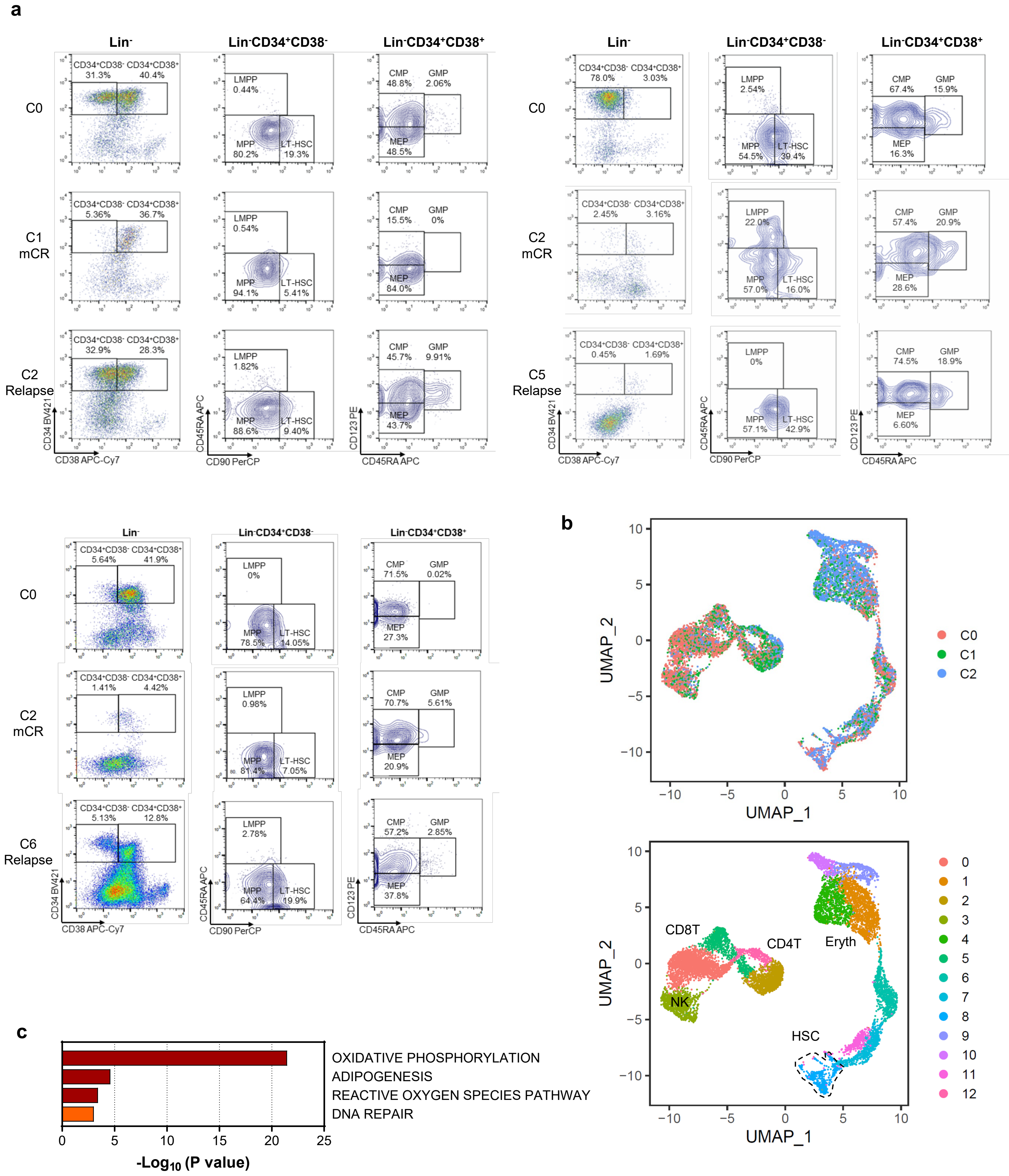

Supplementary Figure 3

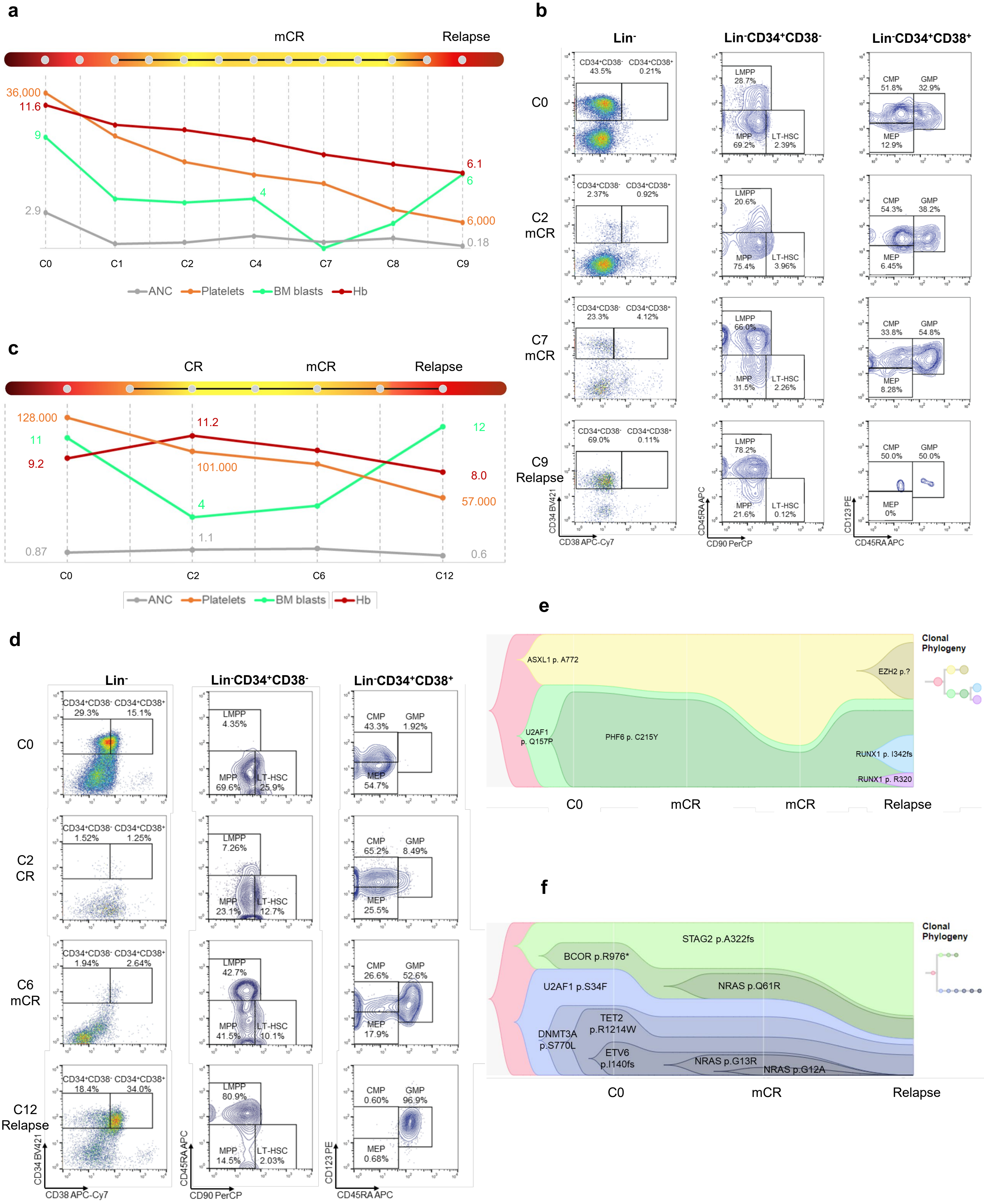

Supplementary Figure 3 (continue)

g

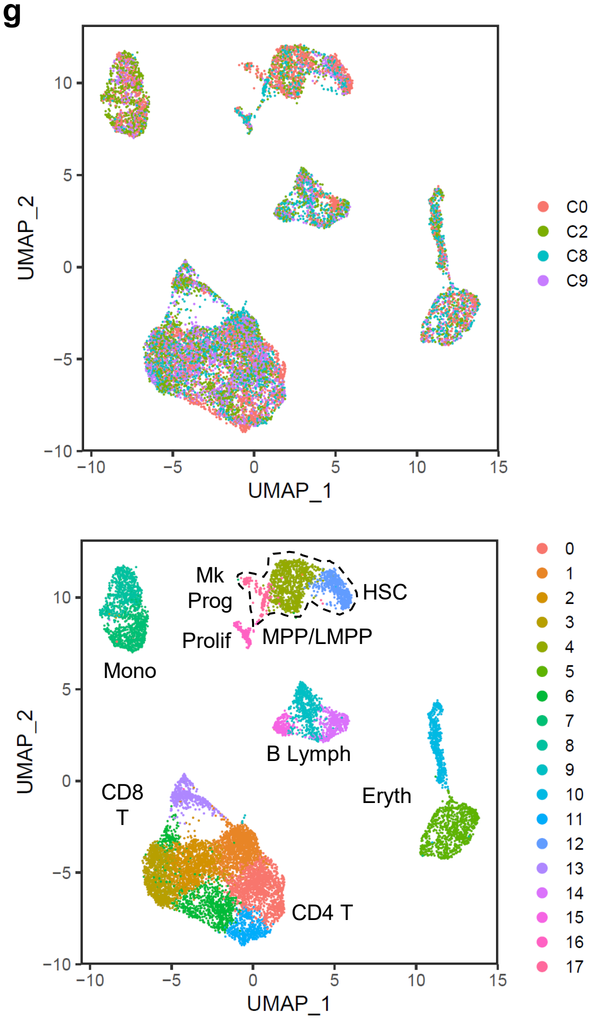

h

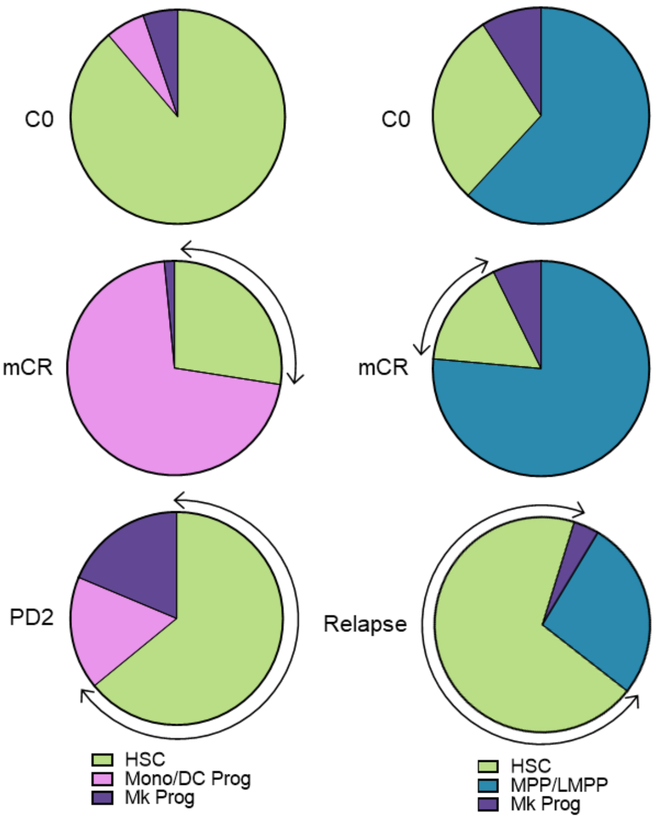

Supplementary Figure 4

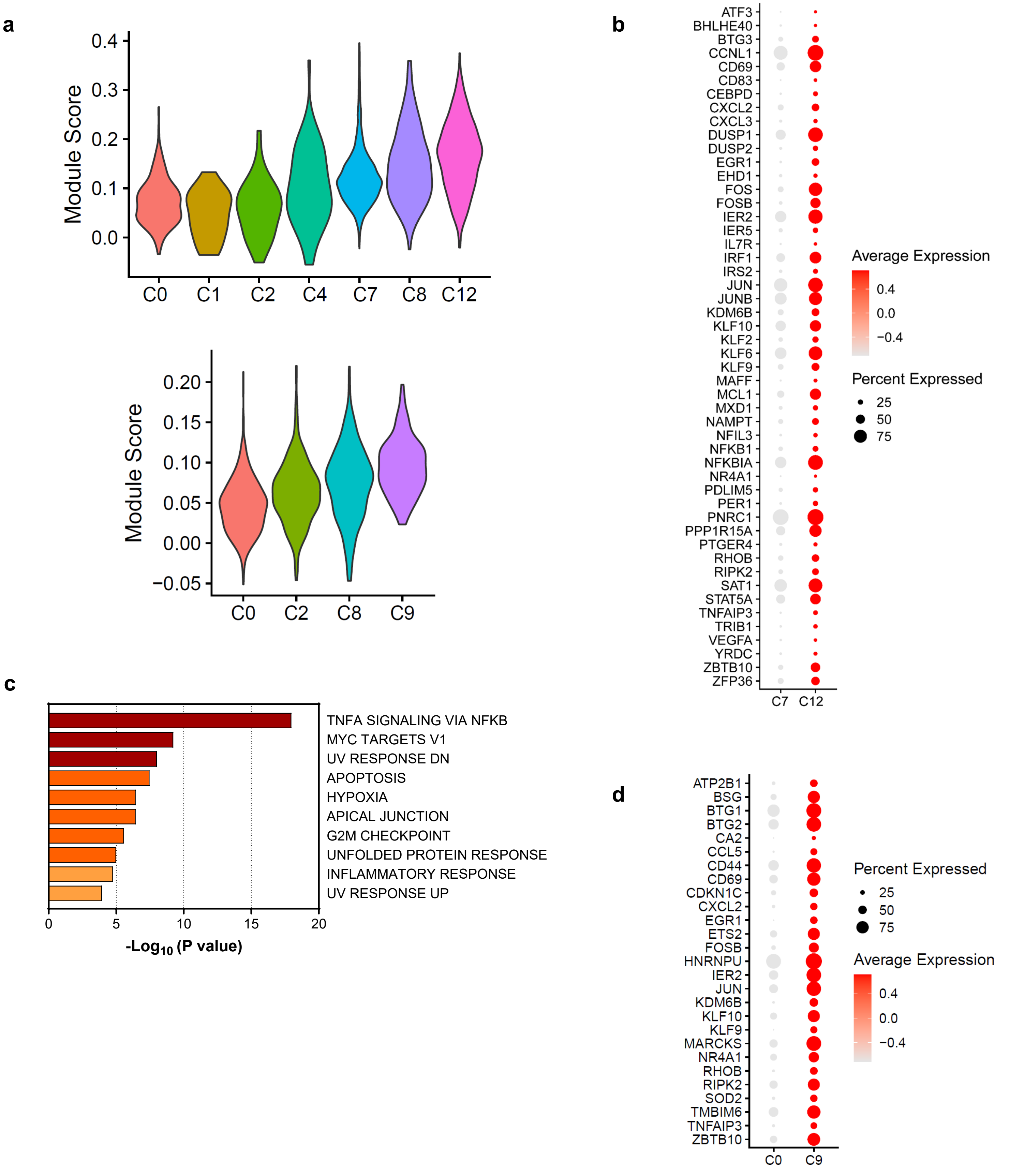

Supplementary Figure 5

a

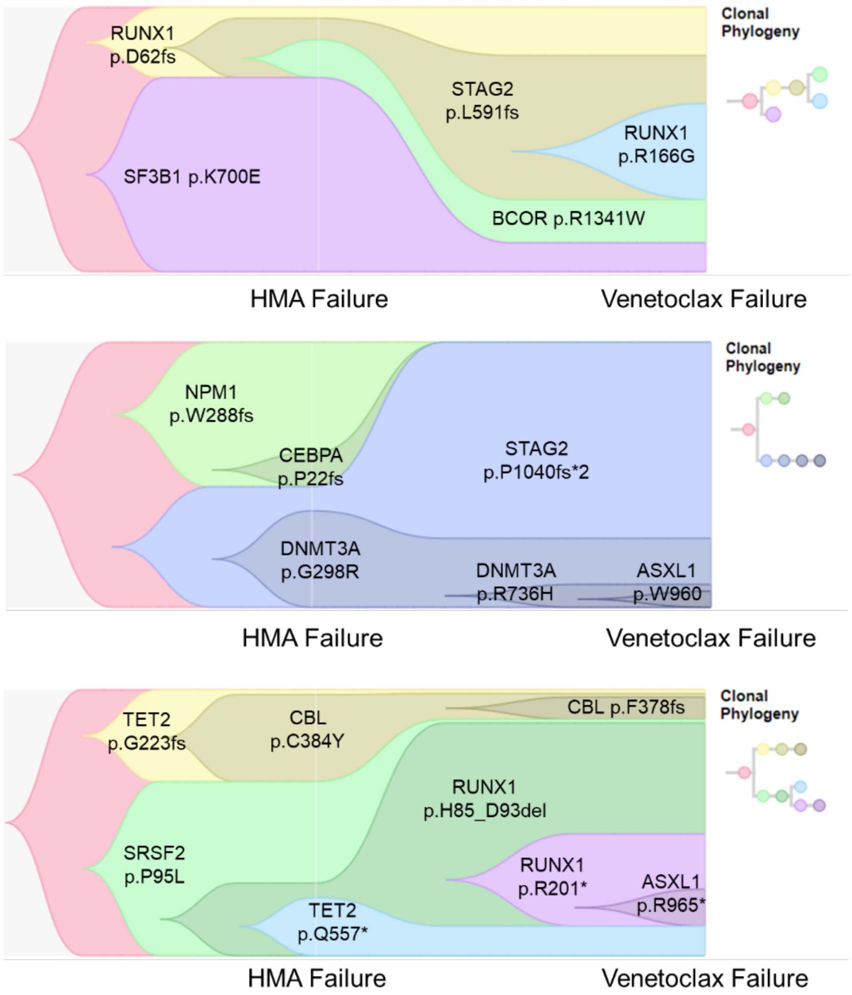

b

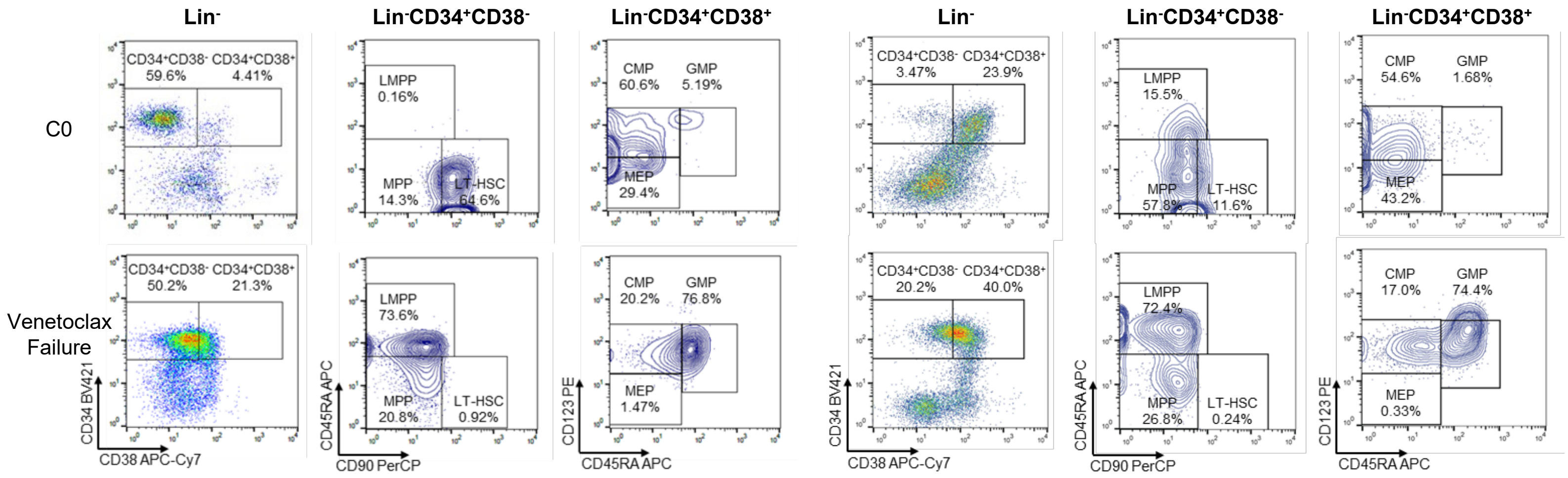

Supplementary Figure 6

a

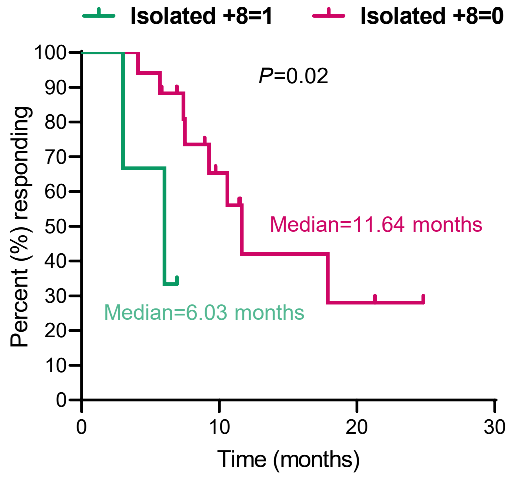

Isolated +8 = 0	18	7	2	0
Isolated +8 = 1	3	0	0	0

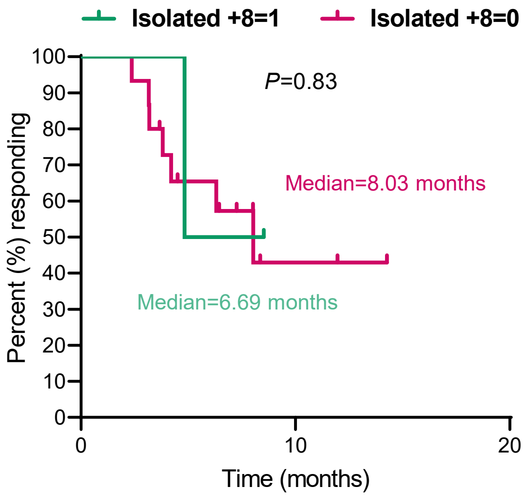

Isolated +8 = 0	18	2	0
Isolated +8 = 1	2	0	0
