# Supplementary material for: Hematopoietic stem cells with granulo-monocytic differentiation state overcome venetoclax sensitivity in patients with myelodysplastic syndromes
Source: Nat Commun. 2024 Mar 18;15:2428. doi: 10.1038/s41467-024-46424-3 (PMC10948794; doi:10.1038/s41467-024-46424-3)
Supplement: Supplementary file 3 — Reporting Summary [file 41467_2024_46424_MOESM3_ESM.pdf]

nature portfolio

Corresponding author(s): Simona Colla

Last updated by author(s): Dec 16, 2023

# Reporting Summary

Nature Portfolio wishes to improve the reproducibility of the work that we publish. This form provides structure for consistency and transparency in reporting. For further information on Nature Portfolio policies, see our Editorial Policies and the Editorial Policy Checklist.

## Statistics

For all statistical analyses, confirm that the following items are present in the figure legend, table legend, main text, or Methods section.

| n/a | Confirmed | |
|---|---|---|
| ☐ | ☒ | The exact sample size (*n*) for each experimental group/condition, given as a discrete number and unit of measurement |
| ☐ | ☒ | A statement on whether measurements were taken from distinct samples or whether the same sample was measured repeatedly |
| ☐ | ☒ | The statistical test(s) used AND whether they are one- or two-sided<br>*Only common tests should be described solely by name; describe more complex techniques in the Methods section.* |
| ☐ | ☒ | A description of all covariates tested |
| ☐ | ☒ | A description of any assumptions or corrections, such as tests of normality and adjustment for multiple comparisons |
| ☐ | ☒ | A full description of the statistical parameters including central tendency (e.g. means) or other basic estimates (e.g. regression coefficient) AND variation (e.g. standard deviation) or associated estimates of uncertainty (e.g. confidence intervals) |
| ☐ | ☒ | For null hypothesis testing, the test statistic (e.g. *F*, *t*, *r*) with confidence intervals, effect sizes, degrees of freedom and *P* value noted<br>*Give P values as exact values whenever suitable.* |
| ☒ | ☐ | For Bayesian analysis, information on the choice of priors and Markov chain Monte Carlo settings |
| ☒ | ☐ | For hierarchical and complex designs, identification of the appropriate level for tests and full reporting of outcomes |
| ☒ | ☐ | Estimates of effect sizes (e.g. Cohen's *d*, Pearson's *r*), indicating how they were calculated |

*Our web collection on statistics for biologists contains articles on many of the points above.*

## Software and code

Policy information about availability of computer code

| | |
|---|---|
| Data collection | BD FACSDiva, version 8.01 (https://www.bdbiosciences.com) |
| Data analysis | FlowJo, version 10.5.3 (www.flowJo.com); GraphPad Prism, version 9 (www.graphpad.com); R, version 4.0.320 (www.r-project.org); Jamovi version 2.0.021 (www.jamovi.org); Metascape (https://metascape.org); Seurat, version 4 (https://satijalab.org/seurat). All softwares are included in the “Methods" section of the paper. |

For manuscripts utilizing custom algorithms or software that are central to the research but not yet described in published literature, software must be made available to editors and reviewers. We strongly encourage code deposition in a community repository (e.g. GitHub). See the Nature Portfolio guidelines for submitting code & software for further information.

## Data

Policy information about availability of data

All manuscripts must include a data availability statement. This statement should provide the following information, where applicable:

- Accession codes, unique identifiers, or web links for publicly available datasets
- A description of any restrictions on data availability
- For clinical datasets or third party data, please ensure that the statement adheres to our policy

Data sets generated in this study using scRNA-seq have been deposited at GEO under accession code GSE241417. Source data are provided as a Source Data file.

## Research involving human participants, their data, or biological material

Policy information about studies with human participants or human data. See also policy information about sex, gender (identity/presentation), and sexual orientation and race, ethnicity and racism.

| | |
|---|---|
| Reporting on sex and gender | We did not perform sex-based or gender based-analyses. Patient samples were selected based on of diagnosis regardless of sex and gender. Males and females had comparable distributions in both cohorts. The sex of all samples is indicated in Supplementary  Table 1. Gender is not relevant to this study because MDS affect both females and males. |
| Reporting on race, ethnicity, or other socially relevant groupings | Patient samples were selected regardless of their race. |
| Population characteristics | Covariate-relevant population characteristics were age, sex, diagnosis and cytogenetic and molecular information. |
| Recruitment | Samples were collected from MDS patients enrolled in the clinical trials trials NCT04160052 (Bazinet et al. 2022), NCT04550442 (Desikan et al. 2022) and NCT04655755 (Bataller et al. 2023). |
| Ethics oversight | All samples were obtained following approval of the corresponding Institutional Review Board at the University of Texas MD Anderson Cancer Center (Houston, TX, USA) and in accordance with the Declaration of Helsinki. |

Note that full information on the approval of the study protocol must also be provided in the manuscript.

# Field-specific reporting

Please select the one below that is the best fit for your research. If you are not sure, read the appropriate sections before making your selection.

☒ Life sciences ☐ Behavioural & social sciences ☐ Ecological, evolutionary & environmental sciences

For a reference copy of the document with all sections, see nature.com/documents/nr-reporting-summary-flat.pdf

# Life sciences study design

All studies must disclose on these points even when the disclosure is negative.

| | |
|---|---|
| Sample size | All available sequential samples from the 28 patients included in the clinical trials described above were included in the analyses. |
| Data exclusions | No data were excluded from the analyses. |
| Replication | Biological replicates could not be possible due to the limited amount of hematopoietic stem cells available from each BM sample. Reproducibility was validated using multiple types of analysis. |
| Randomization | Allocation into experimental groups was based on each patient's diagnosis and treatment. Randomization was not possible. |
| Blinding | The analysis of each experiment was performed blindly. The investigators were blinded to group allocation during data collection. |

# Reporting for specific materials, systems and methods

We require information from authors about some types of materials, experimental systems and methods used in many studies. Here, indicate whether each material, system or method listed is relevant to your study. If you are not sure if a list item applies to your research, read the appropriate section before selecting a response.

### Materials & experimental systems

| n/a | Involved in the study | |
|---|---|---|
| ☐ | ☒ | Antibodies |
| ☒ | ☐ | Eukaryotic cell lines |
| ☒ | ☐ | Palaeontology and archaeology |
| ☒ | ☐ | Animals and other organisms |
| ☐ | ☒ | Clinical data |
| ☒ | ☐ | Dual use research of concern |
| ☒ | ☐ | Plants |

### Methods

| n/a | Involved in the study | |
|---|---|---|
| ☒ | ☐ | ChIP-seq |
| ☐ | ☒ | Flow cytometry |
| ☒ | ☐ | MRI-based neuroimaging |

# Antibodies

| | |
|---|---|
| Antibodies used | Use, Species, Antigen, Conjugate, Clone, Dilution, Supplier, Catalog #<br>Flow cytometry, Human, CD2, FITC, RPA-2.10, 1:20, BD Biosciences, 555326<br>Flow cytometry, Human, CD3, FITC, SK7, 1:10, BD Biosciences, 349201<br>Flow cytometry, Human, CD4, FITC, S3.5, 1:20, Thermo Fisher, MHCD0401<br>Flow cytometry, Human, CD7, FITC, 6B7, 1:20, BioLegend, 343104<br>Flow cytometry, Human, CD10, FITC, SJ5-1B4, 1:20, Leinco Technologies, C139<br>Flow cytometry, Human, CD11b, FITC, ICRF44, 1:20, Thermo Fisher, 11-0118-42<br>Flow cytometry, Human, CD14, FITC, MϕP9, 1:20, BD Biosciences, 347493<br>Flow cytometry, Human, CD19, FITC, SJ25C1, 1:10, BD Biosciences, 340409<br>Flow cytometry, Human, CD20, FITC, 2H7, 1:10, BD Biosciences, 555622<br>Flow cytometry, Human, CD33, FITC, P67.6, 1:20, Thermo Fisher, 11-0337-42<br>Flow cytometry, Human, CD56, FITC, B159, 1:40, BD Biosciences, 562794<br>Flow cytometry, Human, CD235a, FITC, HIR2, 1:40, BD Biosciences, 559943<br>Flow cytometry, Human, CD45RA, APC, HI100, 1:10, Tonbo , 20-0458-T100<br>Flow cytometry, Human, CD34, BV421, 581, 1:20, BD Biosciences, 562577<br>Flow cytometry, Human, CD123, PE, 9F5, 1:20, BD Biosciences, 555644<br>Flow cytometry, Human, CD90, Per-CP, 5E10, 1:10, Thermo Fisher, 45-0909-42<br>Flow cytometry, Human, CD38, APC, HIT2, 1:20, BioLegend, 303534 |
| Validation | Every antibody used in this study had been previously validated by the manufacturer. In addition, all of the antibodies used in the flow cytometry experiments had been previously validated by other groups (Will et al. Blood 2012; Pang et al. Proc Natl Acad Sci USA 2013) and by us (Colla et al. Cancer Cell 2015; Thongon et al. Nat Commun. 2021; Ganan-Gomez et al. Nat Med 2022). |

# Clinical data

Policy information about clinical studies
All manuscripts should comply with the ICMJE guidelines for publication of clinical research and a completed CONSORT checklist must be included with all submissions.

| | |
|---|---|
| Clinical trial registration | The clinical trials are registered at ClinicalTrials.gov (NCT04160052, NCT04550442, or NCT04655755) |
| Study protocol | The clinical protocols used in this study were previously released (NCT04160052, Bazinet et al. 2022; NCT04550442, Desikan et al. 2022; NCT04655755, Bataller et al. 2023. |
| Data collection | Patient simples were collected by the Department of Leukemia at MD Anderson Cancer Center from July 2018 and June 2023. BM aspirations. Flow cytometry, cytogenetic, and genetic analyses were performed at baseline (prior to venetoclax-based therapy) and thereafter, as clinically warranted. |
| Outcomes | Patients' responses were assessed using the modified International Working Group 2006 criteria for MDS (Cheson et al. 2006). Overall response included complete response, marrow complete response, hematological improvement, and a combination of marrow complete response and hematological improvement. |

# Flow Cytometry

## Plots

Confirm that:

☒ The axis labels state the marker and fluorochrome used (e.g. CD4-FITC).

☒ The axis scales are clearly visible. Include numbers along axes only for bottom left plot of group (a 'group' is an analysis of identical markers).

☒ All plots are contour plots with outliers or pseudocolor plots.

☒ A numerical value for number of cells or percentage (with statistics) is provided.

## Methodology

| | |
|---|---|
| Sample preparation | A paragraph describing sample preparation is included in the "Methods" section of the manuscript. Briefly, BM aspirates were collected from each patient following standard clinical procedures. BM mononuclear cells were isolated from each sample using the standard gradient separation approach with Ficoll-Paque PLUS (GE Healthcare Lifesciences, Pittsburgh, PA). For cell sorting of HSPCs, MNCs were pre-enriched using magnetic sorting with microbead kits for the specific antigens (Miltenyi Biotec, San Diego, CA) and further purified by fluorescence-activated cell sorting (FACS). Mononuclear cells or preenriched populations were washed with PBS + 10% FBS and stained with the antibody cocktail described above. |
| Instrument | BD Influx Cell Sorter (BD Biosciences) |
| Software | BD FACSDiva, version 8.01 (https://www.bdbiosciences.com) |
| Cell population abundance | Every cell sorting experiment was performed using a 2-step purification protocol (magnetic bead enrichment followed |

| | |
|---|---|
| Cell population abundance | by FACS). In preliminary validation experiments in which we re-run sorted samples to evaluate if the purification was successful, the purity of double-sorted HSPC populations was over 95%. Given that in the experiments included in the paper (scRNA-seq) we sorted very low numbers of cells from rare HSPC populations (5,000-20,000 cells, depending on the experiment), it was impossible to validate each individual experiment. |
| Gating strategy | Our analyses used a previously validated panel to quantify hematopoietic stem and progenitor cells. The gating strategy was extensively described our previous publications (Ganan-Gomez, Star Protocols, 2022; (Ganan-Gomez, Nature Medicine 2022), so we did not included it again. |

☒ Tick this box to confirm that a figure exemplifying the gating strategy is provided in the Supplementary Information.
