# Supplementary material for: Hematopoietic stem cells with granulo-monocytic differentiation state overcome venetoclax sensitivity in patients with myelodysplastic syndromes
Source: Nat Commun. 2024 Mar 18;15:2428. doi: 10.1038/s41467-024-46424-3 (PMC10948794; doi:10.1038/s41467-024-46424-3)
Supplement: Supplementary file 4 — Description of Additional Supplementary Files [file 41467_2024_46424_MOESM4_ESM.pdf]

Description of Additional Supplementary Files

File Name: Supplementary Data 1

Description: Characteristics of MDS patients treated with venetoclax-based therapy at the time of progression after HMA therapy (n=28).

File Name: Supplementary Data 2

Description: Characteristics of patients with "CMP pattern" MDS (n=10) who failed venetoclaxbased therapy.

File Name: Supplementary Data 3

Description: Characteristics of MDS patients at the time of enrollment in clinical trials of venetoclax-based therapy regardless of prior therapies (n=53)

File Name: Supplementary Data 4

Description: Summary of mutations in patients with secondary (UPN#1, UPN#2, UPN#11) and primary (UPN#8, UPN#9, UPN#12) failure to venetoclax-based therapy
